# Dental Trauma Experience, Attitudes and Trauma Prevention in 11- to 13-Year-Old Lithuanian Schoolchildren

**DOI:** 10.3290/j.ohpd.a43309

**Published:** 2020-07-04

**Authors:** Vaida Zaleckienė, Vytautė Pečiulienė, Jolanta Aleksejūnienė, Saulius Drukteinis, Linas Zaleckas, Vilma Brukienė

**Affiliations:** a Teaching Assistant, Institute of Odontology, Faculty of Medicine, Vilnius University, Lithuania. Methodology, data gathering, statistical analysis, writing the manuscript.; b Professor, Institute of Odontology, Faculty of Medicine, Vilnius University, Lithuania. Methodology, writing the manuscript, supervisor of the study.; c Associate Professor, Department of Oral Health Sciences, Faculty of Dentistry, University of British Columbia, Vancouver, BC, Canada. Statistical analysis, writing the manuscript.; d Associate Professor, Institute of Odontology, Faculty of Medicine, Vilnius University, Lithuania. Data gathering.; e Associate Professor, Institute of Odontology, Faculty of Medicine, Vilnius University, Lithuania. Data gathering.; f Associate Professor, Institute of Odontology, Faculty of Medicine, Vilnius University, Lithuania. Statistical analysis, writing the manuscript.

**Keywords:** dental trauma, children, mouthguards, trauma index, self-reports

## Abstract

**Purpose:**

To identify the prevalence and determinants of dental trauma in permanent anterior teeth among 11- to 13-year-old schoolchildren, to compare self-reported dental trauma rates with clinical examination trauma rates, and to examine mouthguard use among children engaged in contact sports.

**Materials and Methods:**

A probability sampling scheme was used to recruit schools, with all 6th grade children in the selected schools invited to participate. A total of 2621 caregiver consent forms were sent, of which 807 were returned (31%). Caregivers were asked four trauma-related questions. Clinical examinations included assessment of dental trauma experience (trauma index with five severity codes), lip competence and incisal overjet. Child- and tooth-level analyses were performed.

**Results:**

Clinical evidence of dental trauma was found in 52% of participants, 13% self-reported dental trauma, and 7% of children did not remember the occurrence of any trauma. The two most frequently self-reported reasons of dental injury were falls or collisions with objects (63%) and sport/leisure activities (32%). Nearly half of the more severe dental injuries did not receive the necessary treatments. One-third of schoolchildren participated in contact sport activities, but only 3% always used mouthguards. There was a substantial difference between the clinical and self-reported findings. Logistic regression analyses revealed no statistically significant associations between dental trauma experience and the potential determinants studied: gender, lip coverage and overjet.

**Conclusion:**

In this study, traumatic dental injuries were observed with 52% prevalence. A substantial discrepancy between self-reported and clinical findings was observed.

Most frequently children experience traumatic dental injuries (TDI) between 10 and 12 years of age, with prevalence ranging from 4 to 59%.^8,20,23,28^ Traumatic dental injuries have been associated with aesthetic and functional problems; they may also lead to pathological changes in the pulp and periapical region.^7,18,21^ Moreover, different types of treatments to address advanced trauma may be costly, long-lasting and in many cases might require involvement of a multidisciplinary dental team.^11^

Two different approaches to study dental trauma have been used: hospital-based and population-based studies. Hospital-based studies focus not only on visual signs of TDI, but also on severe trauma cases and their complications. However, hospital-based studies might underestimate the overall prevalence of dental traumas.^25^ On the other hand, population-based studies might underreport dental injuries due to the exclusion of radiographic examinations, but their results can be generalised to populations.^2,11,21,30,31^ An important consideration that previous studies identified was that a substantial number of TDI in schoolchildren were either never treated or received inadequate treatments.^19,21,30^ TDI are still a dental public health problem due to their prevalence, inadequate management and the young age of those affected.^6,7,9^

It is important to mention that the clinical diagnoses of dental injuries made by dentists may differ from the children’s and/or their parents’ self-perceptions about the same injury. This difference might be related to the level of seriousness of the specific trauma case. Nevertheless, even minor traumatic injuries such as enamel infraction or fracture can cause pulp necrosis which could lead not only to periapical pathology, but also to arrested root development.^4^

Multiple determinants of traumatic dental injury have been reported, these include: social, cultural, and gender determinants; individual anatomical discrepancies, such as the size of incisal overjet and lip competence; along with failure to use mouthguards.^3,10,12,17,23,25,28,29^ Children who are actively engaged in sports are required to wear mouthguards in order to prevent or to minimise the severity of dental injuries. However, it is well-known that many children participating in sports do not use mouthguards, indicating that a discrepancy between professional advice and its uptake exists.

The aims of the current study were: (1) to identify the prevalence and determinants of dental trauma in permanent anterior teeth among 11- to 13-year-old schoolchildren; (2) to compare self-reported dental trauma rates with clinical examination trauma rates; and (3) to examine mouthguard use among children engaged in contact sports.

## Materials and Methods

The study was approved by the Vilnius Regional Biomedical Research Ethics Committee (158200-2012/11). At the time of the current study there were a total of 4782 6th grade schoolchildren in Vilnius public schools (Department of Statistics, Lithuania). The required minimum sample size of 686 children was based on the 95% confidence interval level, 5% standard error, 90% power, and the expected 23% dental trauma prevalence. From a total of 52 public Vilnius schools, 26 schools were randomly selected. Given that numbers of children in these schools varied, an equal probability sampling scheme was used with a probability of being selected proportional to the school size (ie, if one school had twice as many schoolchildren as another it was given twice the chance of being selected). All 6th grade children in the selected schools were invited to participate. An informed consent form explained the aims of the study.

The questionnaire included the following questions with the corresponding answer choices: Has your child ever suffered an injury to their permanent anterior tooth/teeth? (1) No; (2) don’t remember; or (3) yes. What was the cause of your child’s trauma? (1) Sport or leisure activities; (2) traffic accidents; (3) violence; (4) other accidents with impact with hard surfaces; or (5) other reasons. Does your child participate in contact sport activities? (1) No or (2) yes. Does your child use a mouthguard during sport activities? (1) Always; (2) sometimes; (3) no – but we know about this form of protection; or (4) no – we don’t know about this form of protection.

A total of 2621 caregiver consent forms were sent, of these 807 were returned (response rate, 31%). Due to a child’s refusal to participate or absence from school during a clinical examination, the final sample included a total of 757 children. Dental trauma rates were compared between the self-reported and clinically indicated trauma experiences. Clinical examinations also included the assessment of lip coverage and incisal overjet. All clinical examinations were performed by the same dentist (VZ), who, prior to the study was calibrated with an experienced endodontist specialising in dental traumatology. This calibration involved 50 cases, which were independently examined by both a trainee and an endodontist. The Cohen’s Kappa scores were above 0.84, thus the calibration was considered successful. During data collection, the intraexaminer agreement of clinical assessments could not be assessed because duplicate examinations were not feasible as schools did not permit repeated entries.

Twelve permanent anterior teeth were examined in each child. Prior to a clinical examination, teeth were cleaned of any residual debris and dried. The child’s maxillary/mandibular permanent incisors and canines were examined under natural light, supplemented with artificial lighting, in school rooms during school hours. During this examination the following information was collected: the child’s year of birth and gender; the type of damage sustained; any treatment which had been carried out; which tooth or teeth were involved; the size of the incisal overjet; and lip competence. In addition, photographs of the anterior teeth were taken from the front and profile views in a centric occlusion.

Dental trauma experience was evaluated by the commonly used trauma index (TI)^15,24^: code 0, a tooth free from signs of trauma; code 1, an unrestored enamel fracture or crack; code 2, an untreated fracture that involves dentine; code 3, untreated tooth damage with discolouration and/or the presence of swelling or a fistula; code 4, a missing tooth; and code 5, a restored fracture. Codes 4 and 5 were only assigned if trauma was self-reported.

Visual inspection of the lip’s competence was based on the child’s appearance in the examination room. Competence was designated if the lip covered the maxillary incisors in a resting position, while incompetence was designated if a larger part of the crown height was exposed and visible. Incisal overjet was measured in millimetres using the depth probe of a Vernier calliper from the labioincisal edge of the most prominent maxillary incisor to the labial surface of the corresponding mandibular incisor. This measurement was taken with the teeth in centric occlusion.

Data was analysed with SPSS 25.0 software with the level of statistical significance set at p <0.05.

## Results

Of all those examined, 50% were girls and 50% were boys. Data was analysed at child (n = 757) and tooth (n = 9084) levels.

### Child-level Analyses

Clinical evidence of dental trauma injuries was found in 52% (n = 393) of participants, self-reported dental trauma was indicated by 13% (n = 96) of subjects, and 7% of children could not answer or did not remember. Self-reported dental trauma rates are compared to clinical findings according to the TI codes in Table 1. The overall trend indicated that the worse the trauma, the more thoroughly it was remembered (p <0.001). Self-reported dental trauma was indicated by 15% of boys and 10% of girls, while clinical signs of dental injury were observed in 54% of boys and 50% of girls. The two most frequently self-reported reasons of dental injury were: falls/collisions with objects (63%, n = 60) and sport/leisure activities (32%, n = 31).

**Table 1:** Clinically diagnosed trauma in comparison to self-reports (child-level analyses)

Clinical Codes	Self-reports of dental trauma rates			
	Yes n (%)	No n (%)	Don’t remember n (%)	p values #
Code 0	14 (3.8)	320 (87.9)	30 (8.2)	<0.001
Code 1	29 (8.7)	274 (82.5)	29 (8.7)	0.044
Code 2	13 (50.0)	12 (46.2)	1 (3.8)	<0.001
Code 3	1 (100)	0	0	N/A
Code 4	0	0	0	N/A
Code 5	29 (85.3)	5 (14.7)	0	<0.001
Codes 0–1	43 (6.2)	594 (85.3)	59 (8.5)	<0.001
Codes 2–5	43 (70.5)	17 (27.9)	1 (1.6)	<0.001

# Chi-squared test or Fisher’s exact test

The distribution according to the potential dental trauma determinants is presented in Table 2. There were no statistically significant gender-related differences in dental trauma rates (p <0.177). There was also no statistically significant difference (p = 0.449) in trauma rates among children with ‘competent’ and ‘incompetent’ lip coverage. A reverse overjet (from –0.1 mm to –5.2 mm) was found in eight subjects (1%), none of the children with this type of overjet self-reported any dental trauma, but upon clinical examination trauma was found in three out of eight participants. All injured teeth in these subjects were classified as TI code 1. No statistically significant trauma-related differences were observed among different overjet groups (p <0.326).

**Table 2:** Determinants of dental injuries in 11–13-year-old children (child-level analyses)

Determinants	Clinical signs of dental trauma		p values #
	Absent n (%)	Present n (%)	
Gender			
Boys	172 (47.3)	205 (52.2)	0.177
Girls	192 (52.7)	188 (47.8)	
Lip coverage			
Incompetent	21 (42.9)	28 (57.1)	0.449
Competent	343 (48.4)	365 (51.6)	
Overjet			
<3.6 mm	209 (57.4)	205 (52.2)	0.326
3.6–4.5 mm	78 (21.4)	86 (21.9)	
4.6–5.5 mm	34 (9.3)	55 (14.0)	
5.6–6.5 mm	23 (6.3)	23 (5.9)	
>6.5 mm	20 (5.5)	24 (6.1)	

# Chi-squared test or Fisher’s exact test.

Multiple logistic regression analysis was used to examine potential determinants in regard to dental trauma experience. In preparation for this analysis, five different levels of incisal overjet were recoded into a total of four dummy variables: 0 (<3.6 mm; used as a reference category), 1 (3.6–4.5 mm), 2 (4.6–5.5 mm), 3 (5.6–6.5 mm), and 4 (>6.5 mm). Logistic regression analysis revealed no statistically significant associations between dental trauma experience and any of the three potential determinants, namely, gender, lip coverage and overjet (Table 3).

**Table 3:** Potential determinants of dental trauma injuries (child-level analyses)

Model summary: n = 757; Nagelkarke R square = 0.022; p = 0.318		
Determinants	Odds ratio (95% CI)	p values #
Gender	0.9 (0.5; 1.5)	0.622
Lip coverage	2.8 (0.6; 13.1)	0.199
Overjet groups		
<3.6 mm (reference category)		
3.6–4.5 mm	1.0 (0.5; 2.0)	0.902
4.6–5.5 mm	1.6 (0.8; 3.5)	0.203
5.6–6.5 mm	0.3 (0.1; 2.2)	0.246
>6.5 mm	2.1 (0.7; 6.3)	0.165

# Logistic multiple regression.

Of all the clinically confirmed TI cases (n = 393), 54% children had one traumatised tooth and 31% of children had two teeth that had experienced trauma.

Of all those examined, 210 (30%) schoolchildren participated in contact sport activities (basketball, football, martial arts, etc). Only 3% always used mouthguards, 9% used them occasionally, 76% knew about this type of trauma prevention but did not use them, and 13% had no information about the benefits of mouthguards.

### Tooth-level Analyses

Of the 9084 teeth, 93% didn’t show any signs of dental trauma (code 0). Out of all the teeth with a clinical indication of injury (n = 658), 89% had minor alterations (code 1) such as enamel cracks or fractures limited to the enamel and did not require any restorative treatment (except for aesthetic reasons), 5% had unrestored enamel-dentine defects (code 2), 7% had fractures which were restored with fillings (code 5), and one tooth (0.2%) was diagnosed as a code 3 (untreated traumatic injury with discolouration and fistula). More extensive damage, as indicated by trauma codes 2–5 (treatment needed or performed), was diagnosed in 11% of all teeth that indicated trauma experience. Of all the traumatised teeth with codes 1–5 (n = 658), 5% had not received any professional treatment, but among the more severe injuries (codes 2–5) 41% of cases were untreated.

The teeth most commonly affected by trauma were the maxillary central incisors, no statistically significant difference was observed between the right and left sides. The canines were the teeth least affected (<1%). The distribution of TI codes 2–5, according to tooth type, is illustrated in Table 4, which shows that more severe injuries were mainly diagnosed in the maxillary central incisors while the canines were free from more severe trauma.

**Table 4:** Distribution of dental injuries (TI codes 2–5) in anterior teeth

Tooth 13 n (%)	Tooth 12 n (%)	Tooth 11 n (%)	Tooth 21 n (%)	Tooth 22 n (%)	Tooth 23 n (%)
0 (0.0)	7 (9.3)	26 (34.7)	19 (25.3)	2 (2.7)	0 (0.0)
Tooth 43 n (%)	Tooth 42 n (%)	Tooth 41 n (%)	Tooth 41 n (%)	Tooth 42 n (%)	Tooth 43 n (%)
0 (0.0)	2 (2.7)	10 (13.3)	4 (5.3)	5 (6.7)	0 (0.0)

## Discussion

Substantial inconsistencies were found between self-reported and clinically observed signs of dental injuries, with instances identified almost four times more frequently with a clinical examination as compared to those indicated in self-reports. This finding indicates a lack of knowledge about the consequences of untreated dental trauma among this population of Lithuanian children, possibly among their caregivers as well. This finding is in accordance with other studies and might be explained by the fact that some children and parents are not worried about minor TDI such as enamel cracks or small fractures, possibly because of the negligible impact on esthetics.^5,25^ The fact that one-fifth of self-reported trauma cases lacked clinical signs of dental injury might be related to a different type of dental injury, for example, one that affected the periodontal or root tissues and could not be detected by the clinical examination employed in the present study. Caregivers of children indicating experience of more severe trauma (codes 2 and 3) received a referral letter inviting them to a free specialist consultation; surprisingly, none of the invitations were accepted.

The present study found the prevalence of clinically confirmed dental trauma in 52% of the 11- to 13-year-old Lithuanians studied. Due to ethical and feasibility issues, radiographic examinations were not included, consequently a higher rate of TDI could be expected.^2,23^ In other studies, the prevalence of trauma to the upper central incisors ranged from 32% to 92%,^6,9,21,28^ while in our study trauma to the maxillary central incisors constituted almost half of all the trauma cases.

In previous studies, dental trauma prevalence was reported up to 60%.^3,6,20,26^ Prevalence varies among different countries; in addition that different prevalence rates exist between reports from the same country, for example, in Brazil it varied from 10% to 59%.^20,28,30^ The reasons behind such variation might be due to differences in sampling, diagnostic criteria and other methodological aspects.^5^ Therefore, caution is recommended when comparing dental trauma prevalence rates among various studies.^31^

The current study reports higher rates of dental trauma experience in Lithuanian children than in other countries, possibly due to differences in the diagnostic criteria chosen.^5,11,23^ In our study, all types of dental injuries including enamel cracks and fractures were recorded, while all types of dental injuries are usually not reported in other studies.^23,24^ Since enamel cracks and small fractures without dentine involvement comprised nearly 90% of all clinically observed signs of dental trauma in our study, we would argue that the exclusion of minor injuries might underestimate the overall prevalence of dental traumas. Our rates are slightly higher than reported in previous studies, where uncomplicated enamel fracture prevalence worldwide varied from 24%-83%.^5,16,21^ On the other hand, some reports showed almost equal distribution of enamel and enamel-dentine fractures.^18,29^ In the current study, untreated enamel-dentine fractures constituted almost 5% of all lesions – which is similar to the Fakhruddin et al and Malikaew studies.^8,16^ Other studies reported a higher prevalence of moderate injuries, ranging from 9% to 23%.^5,22^ In the present study, 93% of dental traumas were left untreated; our findings are in accordance with other studies.^13,20,21^ The majority of these dental traumas were of low severity, consequently they did not require any dental treatment. Concomitantly, treatment negligence was commonly observed (41%) among the cases where treatments were required (TI codes 2–5). Limited access to professional dental care, a lack of dental awareness, and dental treatment costs are known reasons for traumatised teeth being left untreated.^13^ Also, it could be due to the fact that minor traumas provoke short-duration symptoms which quickly disappear, consequently parents may not be aware the trauma occurred. Caregivers and their children may also lack knowledge about the possible consequences of traumatic injuries, such as pulp necrosis.^4^ Our study found that the proportion of traumatised teeth receiving treatments (7%) was relatively low in comparison to other studies reporting treatment frequencies of 13–28%.^3,13,22,30^

Differences in reports about social and cultural determinants exist between countries. The present study found that falls and collisions with objects were the most common reasons for dental injuries. This finding is in accordance with many previous reports, while studies in Brazil and Syria showed that violence and traffic accidents were the leading causes.^17,18,28,31^ Concurrently, we need to consider that some causes of dental traumas (eg, those related to domestic violence) might be unreported due to fear or shame, or that these cases might instead be reported as falls, collisions or as an unknown cause.^18^

Dental trauma due to participation in sporting activities can be prevented with mouthguards or extraoral protective devices.^4^ Unfortunately, the measures needed for trauma prevention are usually only implemented after trauma occurs. In our study, the majority of children participating in contact sports had knowledge about mouthguards, but only a very small proportion of them were wearing it on a regular basis. A lack of such prevention was also reported in Jordan and Israel.^1,14^

We did not identify any statistically significant determinants of dental injuries in the multivariate analyses. It is possible that gender-related differences in trauma rates were not found because growing numbers of girls enrol in sports and some boys prefer computer-based activities, leading to reduced time for boys to engage in daily physical activity.^11,30^ Other studies observed more frequent dental trauma cases in males than in females.^12,17,19,23,29^

The reported risk threshold for an increased overjet varied from 3 mm to ≥5 mm in different studies.^10,28^ The current study did not find a statistically significant relationship among overjet and tooth trauma in contrast to other studies.^3,12,19,27^ In our study, children with inadequate lip coverage experienced similar trauma rates as those with adequate lip coverage; this finding was also in contrast with several studies that reported a protective effect of adequate lip coverage on dental traumas.^19,20,30^

## Conclusions

The prevalence of TDI among Lithuanian elementary school-age children was high, as more than half of the children presented with clinical signs of dental injury. A substantial discrepancy between self-reported and objectively observed dental injuries was identified. The majority of dental traumas observed were minor enamel injuries that did not require any dental treatment. Concomitantly, nearly half of the more severe dental injuries did not receive the necessary treatments.
